# 20(*S*)-Protopanaxadiol Inhibits Angiotensin II-Induced Epithelial- Mesenchymal Transition by Downregulating SIRT1

**DOI:** 10.3389/fphar.2019.00475

**Published:** 2019-05-07

**Authors:** Yuchen Wang, Huali Xu, Wenwen Fu, Zeyuan Lu, Minyu Guo, Xueji Wu, Mingyang Sun, Yanzhe Liu, Xiaofeng Yu, Dayun Sui

**Affiliations:** Department of Pharmacology, School of Pharmaceutical Sciences, Jilin University, Changchun, China

**Keywords:** 20(*S*)-protopanaxadiol, angiotensin II, non-small cell lung cancer, epithelial-mesenchymal transition, SIRT1

## Abstract

20(*S*)-Protopanaxadiol (PPD) is one of the major active metabolites in ginseng saponin. Our previous studies revealed a broad spectrum of antitumor effects of PPD. Angiotensin II (Ang II), the biologically active peptide of the renin-angiotensin system (RAS), plays a critical role in the metastasis of various cancers. However, its role in the anti-metastatic effects of PPD is not clearly understood. In this study, we investigated the inhibitory effect of PPD on Ang II-induced epithelial-mesenchymal transition (EMT) in non-small cell lung cancer (NSCLC) cells, and the potential molecular mechanisms of suppression of NSCLC migration and metastasis by PPD. Treatment of A549 cells with Ang II increased metastases in an experimental model of cancer metastasis *in vivo*. PPD effectively prevented Ang II-induced EMT, as indicated by upregulation of E-cadherin and downregulation of vimentin. Additionally, Ang II upregulated the class III deacetylase sirtuin 1 (SIRT1) expression in EMT progression, while downregulation of SIRT1 was involved in suppression of Ang II-induced EMT by PPD. Moreover, the inhibitory effect of PPD was reversed by SIRT1 upregulation, and PPD demonstrated synergy with an SIRT1 inhibitor on Ang II-induced EMT. Taken together, our data reveal the mechanism of the anti-metastatic effects of PPD on Ang II-induced EMT and indicate that PPD can be used as an effective anti-tumor treatment.

## Introduction

Lung cancer is a leading cause of death in both men and women, with over 1,000,000 new cases diagnosed worldwide annually and a 5-year survival rate of only 14% (Chen et al., 2016; Siegel et al., 2018). Advanced stage metastasis is the major cause of the high mortality at initial diagnosis. During lung carcinogenesis, epithelial-mesenchymal transition (EMT) is involved in non-small cell lung cancer (NSCLC) progression through several etiologies and mechanisms to promote tumor cell invasion and metastasis (Zeisberg and Neilson, 2009; Bartis et al., 2014). During metastasis progression, cancer cells gain access to the blood and lymphatic systems and metastasize to distant sites in the body (Zeisberg and Neilson, 2009; Bartis et al., 2014).

Angiotensin II (Ang II), a primary active peptide of the renin-angiotensin system (RAS), regulates cell proliferation, alterations of fibrosis, and vascular permeability (Hsueh et al., 1995; Nouet and Nahmias, 2000; Iwai and Horiuchi, 2009). Recently, Ang II was reported to play a critical role in promoting tumor cell proliferation, migration, and invasion, including ovarian carcinomas (Suganuma et al., 2005), hepatocellular carcinomas (Xu et al., 2017), and breast cancer (Rodrigues-Ferreira et al., 2012). Additionally, both ACE inhibitors or angiotensin receptor blockers effectively suppress NSCLC cell proliferation as well as metastases to prevent further tumor formation (Maluccio et al., 2001; Attoub et al., 2008). Although Ang II was suggested to be involved in lung cancer proliferation, migration, and invasion processes (Gallagher et al., 2011), few studies have examined the role of Ang II in promoting NSCLC cell EMT.

Recent studies confirmed that the expression of SIRT1, a class III histone deacetylase, regulates the migration of various cancer cells *in vitro* and tumor metastasis *in vivo*, including in hepatocellular carcinoma (Hao et al., 2014), colorectal cancer (Diesch et al., 2014), and gastric cancer (Zhang L. et al., 2013). Specifically, in NSCLC, SIRT1 is highly expressed in tumor issues and is tightly associated with the poor overall survival of patients with NSCLC (Noh et al., 2013). Moreover, SIRT1 is related to the histological grade of patients with NSCLC (Chen et al., 2017). Numerous studies have demonstrated that SIRT1 is related to the aggressiveness of NSCLC cells and overexpression of SIRT1 leads to EMT (Cha et al., 2016). In this study, we explored the involvement of SIRT1 in Ang II-induced EMT during the migration and metastases of NSCLC cells.

Panax ginseng has various pharmacological activities such as anti-inflammatory and anti-cancer effects and has been mainly used as a medicinal plant in Asian regions for millennia (Tian et al., 2016; Wang et al., 2018). Ginsenosides are the main bioactive components. 20(*S*)-protopanaxadiol (PPD) is the final metabolite of protopanaxadiol-type ginsenosides (Kim, 2012) and has a wide range of bioactive effects in the human body (Han et al., 2017; Lu et al., 2018; Zhang et al., 2018). Recent studies reported that PPD inhibits the progression of some types of cancer cells, including lung and breast carcinoma cells (Zhang Y. L. et al., 2013, 2018), but the anti-metastasis effects of PPD remain unclear. Moreover, our previous study showed that the mechanisms of the cardioprotective effects of Rb1 and Rg3 may be related to the attenuation of RAS activity in the myocardium (Jiang et al., 2017). Whether PPD inhibits NSCLC metastasis induced by Ang II is unclear.

In this study, we evaluated the anti-metastatis effect of PPD on Ang II-induced EMT and determined the molecular mechanism of these effects. We showed that PPD inhibited Ang II-induced EMT and suppressed NSCLC cell migration and metastasis *in vivo* and *in vitro*, and that SIRT1 is involved in this process.

## Materials and Methods

### Cell Culture and Reagents

Hainan Asia Pharmaceutical Co., Ltd. (Haikou, China) provided PPD, which had a purity of > 95% as determined by high-performance liquid chromatography. A549 cells and H460 cells were obtained from American Type Culture Collection (Manassas, VA, United States) and cultured at 37°C in a humidified atmosphere of 5% CO_2_ with RPMI-1640 medium, which was supplemented with 10% fetal bovine serum, 100 U/mL penicillin, and 100 mg/mL streptomycin. Ang II and EX-527 were purchased from Sigma-Aldrich (St. Louis, MO, United States). The BCA protein assay reagent kit and DAPI staining kit were purchased from Beyotime Institute of Biotechnology (Jiangsu, China). The primary antibodies for E-cadherin, vimentin, and SIRT1 were purchased from Abcam (Cambridge, United Kingdom). Slug and ZEB1 were purchased from Cell Signaling Technology (Danvers, MA, United States). GAPDH was purchased from ZSGB Biotechnology Co., Ltd. (Beijing, China). The secondary antibodies were purchased from Beijing Dingguo Changsheng Biotechnology Co., Ltd. (Beijing, China).

### Cell Migration Aassays

For the wound-healing assay, A549 cells and H460 cells were seeded into each well of a 6-well plate at a density of 3 × 10^5^ cells/mL. The wounds were made by scratching with a pipette tip. The cells were washed with PBS and then exposed to 20 μM PPD and 500 μM Ang II in RPMI-1640 medium for 24 h. Images were acquired at 0 and 24 h. Quantitation was done by measuring the migrating wound healing area and compared to the start wound area from three independent experiments.

### Transwell Assay

A549 cells and H460 cells were seeded into 24-well-Transwell inserts (Greiner Bio-One, Frickenhausen, Germany) at a density of 4 × 10^4^ cells and the bottom of the inserts was filled with 500 μL medium containing 10% fetal bovine serum and 20 μM PPD. After 3 h incubation, Ang II was added to the bottom of the inserts to a final concentration of 500 nM. For the Transwell invasion assay, the filter in the insert was precoated with Matrigel (BD Biosciences, San Jose, CA, United States). Cells were cultured for 24 h. Cells remaining on the top of the filter membrane were removed using a cotton swab, while cells migrated or invaded across the filter membrane were washed with PBS, fixed with 4% paraformaldehyde for 15 min, and stained with crystal violet solution for 10 min. Images were acquired, and the cells were counted under a Nikon TE-2000U fluorescence microscope (magnification, × 100, Tokyo, Japan).

### Quantitative Real-Time PCR

Cells with different treatment were harvested and total RNA was isolated using Trizol reagent (Invitrogen Inc., Carlsbad, CA) according to the manufacturer’s instructions. RNA was quantitated by optical density measurements at 260 and 280 nm. Complementary DNA synthesis and qPCR were performed by using a TransScript^®^ Green Two-Step qRT-PCR SuperMix (TransGen Biotech, China). QPCR was performed with a reaction mixture (total volume 20 μL) that consisted of 2 × Trans Start Top Green qPCR SuperMix, Passive Reference Dye, ddH2O, cDNA templates, and forward and reverse primers. The amount of Snail, Slug, and Zeb1 mRNA was normalized to GAPDH expression. All of the primers were either ordered or custom made from Beijing Dingguo Changsheng Biotechnology Co., Ltd. (Supplementary Table S1). Relative fold changes in the expression of the target gene in control and other groups were determined using the 2 ^–Δ⁢Δ⁢CT^ method.

### Western Blotting

Western blotting was performed to assess protein expression. Following treatment with PPD, the cells were harvested and lysed in radioimmunoprecipitation assay buffer (Beyotime Biotechnology) for 30 min on ice. The protein concentration was determined using a BCA protein assay kit according to the manufacturer’s protocol. Proteins (20 μL) were separated by 12% SDS-PAGE and transferred onto a polyvinylidene fluoride membrane, which was subsequently blocked with 5% (w/v) non-fat milk for 1 h at room temperature. Membranes were incubated with the appropriate primary antibodies against E-cadherin, vimentin, SIRT1, Slug, ZEB1, and GAPDH at a 1:1,000 dilution at 4°C overnight. Primary antibody binding was detected by incubation with a secondary antibody conjugated to horseradish peroxidase. The goat-anti-mouse and goat-anti-rabbit secondary antibodies (Beijing Dingguo Changsheng Biotechnology Co., Ltd.) were used at 1:5,000 dilution at room temperature for 1 h. The bands were visualized using a BeyoECL Plus enhanced chemiluminescence kit (Beyotime Institute of Biotechnology). ImageJ software (version 1.5.0.26; National Institutes of Health, Bethesda, MD, United States) was used for analysis.

### Immunofluorescence Staining

Cells were cultured on cover slips with Ang II and with or without PPD for 24 h. Cells were then washed with PBS, fixed with 4% paraformaldehyde for 15 min, permeabilized with 0.3% Triton-X for 15 min, and blocked with 5% normal goat serum at room temperature for 1 h. The cover slips were incubated with rabbit polyclonal SIRT1 antibody overnight at 4°C. The cells were washed with PBS and incubated with a fluorescence secondary antibody at room temperature for 1 h. After washing with PBS, the cells were stained with DAPI for 5 min and reviewed, and images were acquired with a Nikon TE-2000U fluorescence microscope (magnification, × 400).

### Tail Vein Injection Lung Cancer Models

Tail vein injection lung cancer metastasis models were generated as described previously (Rodrigues-Ferreira et al., 2012; Lin et al., 2016). Briefly, A549 cells expressing luciferase were treated with 500 nM Ang II (Sigma) or vehicle in medium for 48 h prior to injection. For intravenous injections, 1 × 10^6^ cells were suspended in 0.1 mL of PBS and injected into the tail vein of male nude mice. The mice were anesthetized by exposure to 3% isoflurane and intraperitoneal injection of D-luciferin every 7 days for up to 28 days. After 5 min, the lung metastasis formation of A549 cells was monitored using the IVIS bioluminescent imaging system (PerkinElmer, Waltham, MA, United States).

Nude mice were orally administered PPD for 4 weeks and sacrificed at the end of experiment, after which their lungs were removed and fixed in 10% formalin.

Animals were treated according to the Guide for the Care and Use of Laboratory Animals [United States National Institutes of Health (NIH)] and the Committee for the Care and Use of Laboratory Animals of Jilin University (Changchun, China). The study protocol was approved by the Ethics Committee of Jilin University. Male nude mice were provided by the Experimental Animal Center of Jilin University.

### Statistical Analysis

All data are represented as the mean ± standard deviations for three independent experiments. One-way analysis of variance or Student’s *t* test were used to evaluate statistical differences. The statistically significant standard was *P* values of < 0.05.

## Results

### Ang II Promoted EMT in NSCLC Cells

**FIGURE 1:**
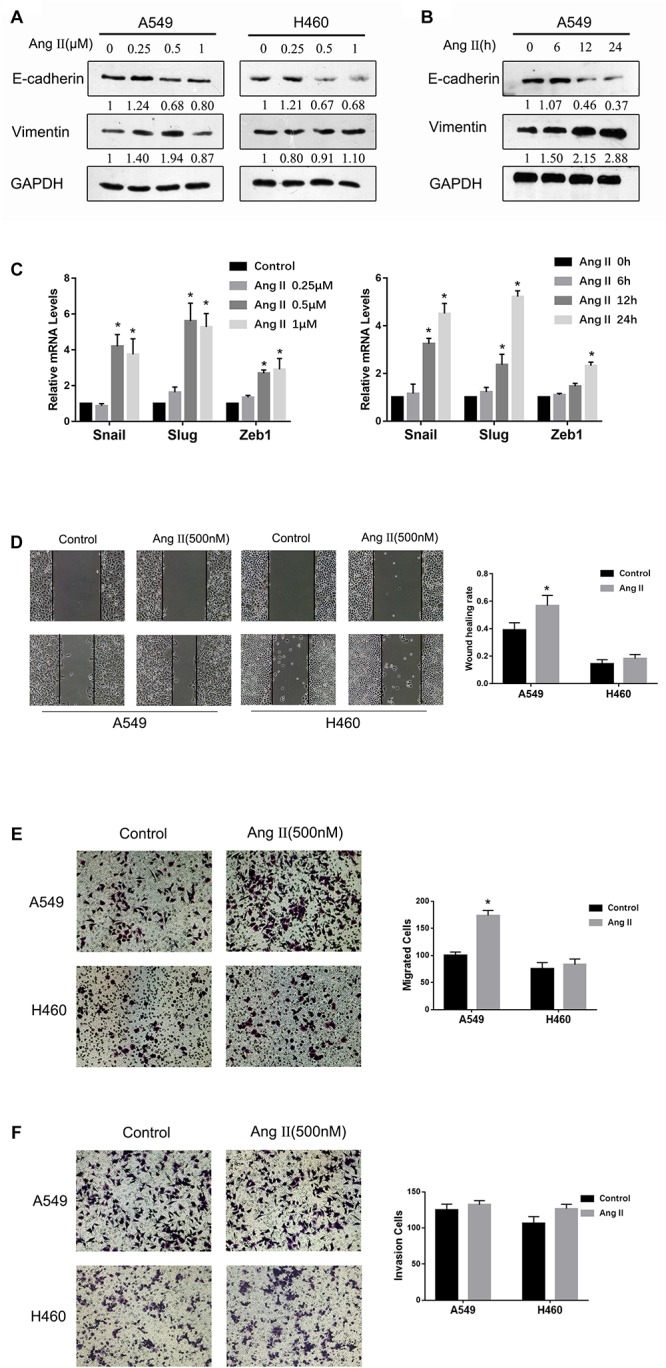
Ang II induces EMT and increases motility of NSCLC cells. **(A)** A549 cells and H460 cells were treated with 0.25, 0.5, or 1 μM Ang II for 24 h and subjected to western blot analysis of E-cadherin and vimentin. GAPDH was used as a loading control. **(B)** A549 cells were treated with 0.5 μM Ang II for 6, 12, or 24 h and subjected to western blot analysis of different proteins. **(C)** The mRNA levels of Snail, Slug and Zeb1 in the A549 cells were measured after Ang II treatment at different time and concentration. **(D)** A549 cells and H460 cells incubated with or without 0.5 μM Ang II for 24 h and subjected to the wound-healing assay to assess tumor cell migration. Images were acquired at 0 and 24 h. **(E)** and **(F)**, Transwell assays assessed tumor cell migration and invasion capacity in A549 cells and H460 cells incubated with or without 0.5 μM Ang II for 24 h. Error bar, SD of three independent experiments. *p < 0.05.

Recent studies showed that ACE inhibitors or ARBs effectively inhibited NSCLC cell proliferation and lung tumor metastases to prevent distal metastasis of the tumor (Attoub et al., 2008). Ang II facilitates the migration and invasion of several types of cancer cells by mediating EMT (Okamoto et al., 2012; Zhao et al., 2014). To assess the direct impact of Ang II on EMT in NSCLC cells, we initially examined the levels EMT biomarkers in A549 cells and H460 cells after Ang II treatment. As shown in Figures 1A,B, Ang II stimulation for 24 h significantly increased E-cadherin expression and decreased vimentin expression in A549 cells. However, treatment with higher doses of Ang II did not show a stronger effect in promoting EMT. The expression of the genes associated with EMT (Snail, Slug, ZEB1 was increased after Ang II stimulation (Figure 1C). The effects of Ang II on cell migration and invasion was evaluated by wound-healing and Transwell assays. As shown in Figures 1D–F, Ang II treatment markedly promoted the migration of A549 cells and showed limited effects on promoting invasion. However, as shown in Figure 1A, Ang II did not show obvious promoting effects on EMT in H460 cells. The changes in E-cadherin and vimentin expression were not significant, and the results of the wound-healing and Transwell assays were negative (Figures 1D–F). Inconsistent results were obtained likely due to Ang II did not increase TGF-β expression on H460 cells (Supplementary Figure S1). Collectively, these results support that Ang II directly promotes the EMT and subsequently enhances lung tumor migration.

### Ang II Promotes A549 Cell Metastasis *in viv*o

**FIGURE 2:**
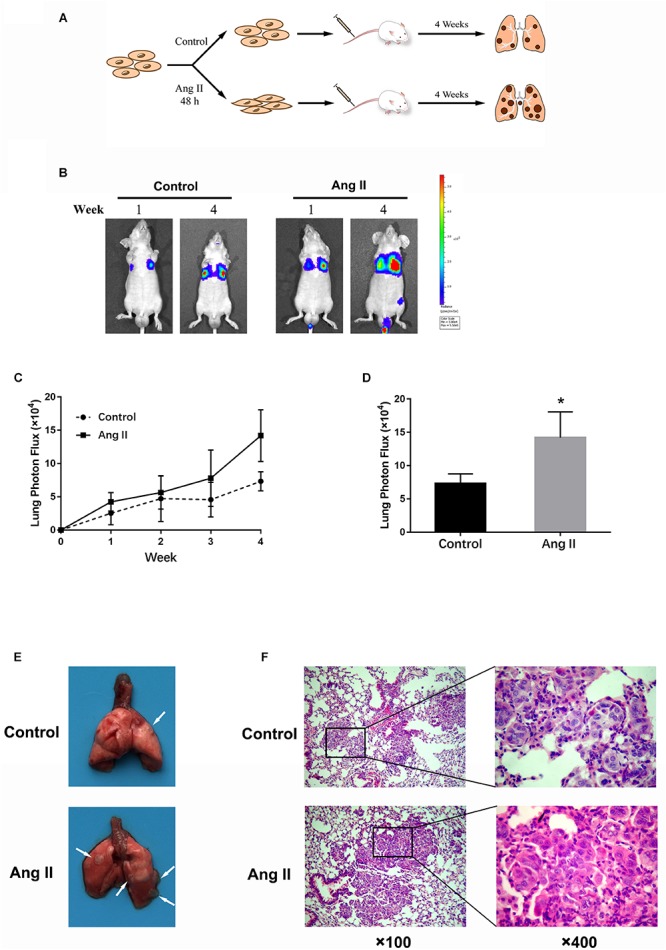
Ang II promotes NSCLC cell metastasis *in vivo*. **(A)** Schema of experimental protocol. Nude mice were intravenously injected with A549 cells pretreated with or without Ang II, as described in the Materials and Methods. After 4 weeks, mice were sacrificed, and lungs were imaged. **(B)** After injection at weeks 1 and 4, the mice were intraperitoneally injected with D-Luciferin for *in vivo* bioluminescence imaging. Tumor metastasis to lungs was shown. **(C)** Mean bioluminescence/time of lung metastasis in xenografted mice, graphed as normalized photon flux/time. **(D)** Mean bioluminescence at 4 weeks. **(E)** Representative images of lung metastatic nodules (arrows indicate tumor lesions). **(F)** Representative pictures of HE staining of the lung issue are shown (magnification, left × 100 and right × 400). *p < 0.05.

To determine whether Ang II promotes NSCLC metastasis *in vivo*, we investigated lung nodule formation in the mouse tail intravenously injected with Ang II-pretreated A549 cells (Figure 2A). The animals were monitored with an *in vivo* imaging system following d-luciferin injection. Ang II-treated cells exhibited lung tumor formation as measured by tumor bioluminescence at week one of the experiment compared to mock-treated cells (Figures 2B–D). At week four, we observed significant expansion of lung metastases in the animals injected with Ang II-pretreated cells (Figure 2B). The mice injected with Ang II-pretreated cells displayed more nodules than the control group and histological analysis of the lung confirmed the presence of tumor cells in the lung samples on the last day of the experiment (Figures 2E,F). Our results indicate that A549 cells show increased metastatic potential *in vivo* after Ang II treatment.

### Ang II Enhanced the Expression of SIRT1

**FIGURE 3:**
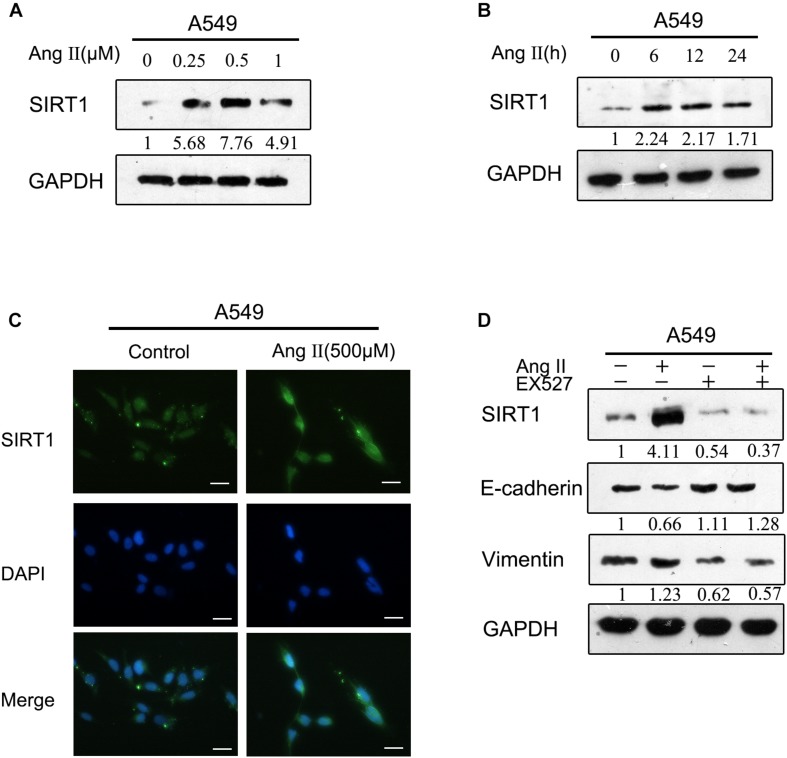
Ang II induces the expression of SIRT1 during EMT. **(A)** and **(B)**. A549 cells were treated with Ang II, and the expression of E-cadherin, vimentin, and SIRT1 was determined by western blotting. **(C)** After treatment with Ang II, A549 cell morphology was examined, and the cells were fixed, permeabilized, and stained with anti-SIRT1 polyclonal antibody (green) and DAPI (blue). Cells were analyzed by fluorescence microscopy. All scale bars represent 25 μm. **(D)** A549 cells were treated with Ang II, with or without EX-527 for 24 h, and then E-cadherin, vimentin, and SIRT1 protein levels were examined by western blotting. Similar data were obtained from three independent experiments.

To improve the understanding of the mechanism of Ang II-induced EMT, we investigated whether SIRT1 is regulated by Ang II. We verified that the expression of SIRT1 was greatly increased after treatment with Ang II in a dose- and time-dependent manner according to western blotting (Figures 3A,B). We also confirmed by immunofluorescence that Ang II induced SIRT1 expression in a dose-dependent manner (Figure 3C). Additionally, EX-527, a selective inhibitor of SIRT1, reversed the EMT marker changes induced by Ang II, suggesting that SIRT1 is an essential regulator of Ang II-induced EMT (Figure 3D).

### PPD Inhibit Ang II-Induced EMT

**FIGURE 4:**
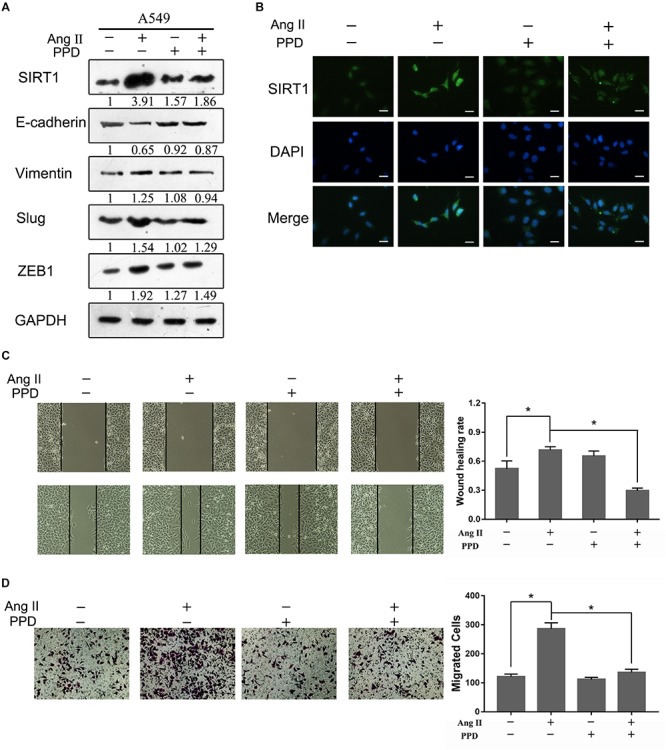
Effect of PPD on Ang II induced EMT and migration in A549 cells. **(A)** A549 cells were treated with Ang II, with or without PPD for 24 h, and the expression of E-cadherin, vimentin, Slug, ZEB1 and SIRT1 was determined by western blotting. **(B)** Cells were fixed, permeabilized, and stained with anti-SIRT1 polyclonal antibody (green) and DAPI (blue). Cells were analyzed by fluorescence microscopy. All scale bars represent 25 μm. **(C)** and **(D)**, Wound-healing assay and Transwell assays assessed tumor cell migration capacity in A549 cells treated with Ang II, with or without PPD for 24 h. Similar data were obtained from three independent experiments. *p < 0.05.

Our previous study showed that the mechanisms of the cardioprotective effects of Rb1 and Rg3 may be related to attenuation of RAS activity in the myocardium (Jiang et al., 2017). Further, PPD did not induce any significant changes in EMT markers in A549 cells (Figure 4A). Therefore, PPD may inhibit EMT markers of Ang II-treated A549 cells. We next examined the effect of PPD on EMT in human NSCLC cells by western blotting. As shown in Figure 4A, 20 μM PPD reversed the decrease in E-cadherin expression and increase in vimentin induced by Ang II. Notably, PPD did not alter SIRT1 expression on A549 cells, but decreased SIRT1 expression as observed by western blotting and immunofluorescence assays (Figures 4A,B). PPD also inhibited the Ang II-induced increase in the expression of Slug and ZEB1 (Figure 4A). The inhibition effect of PPD on cell migration was evaluated by wound healing and Transwell assays. As shown in Figures 4C,D, the results showed that PPD treatment markedly suppressed Ang II induced migration of A549 cells.

### PPD Inhibits Cancer Metastasis *in viv*o

**FIGURE 5:**
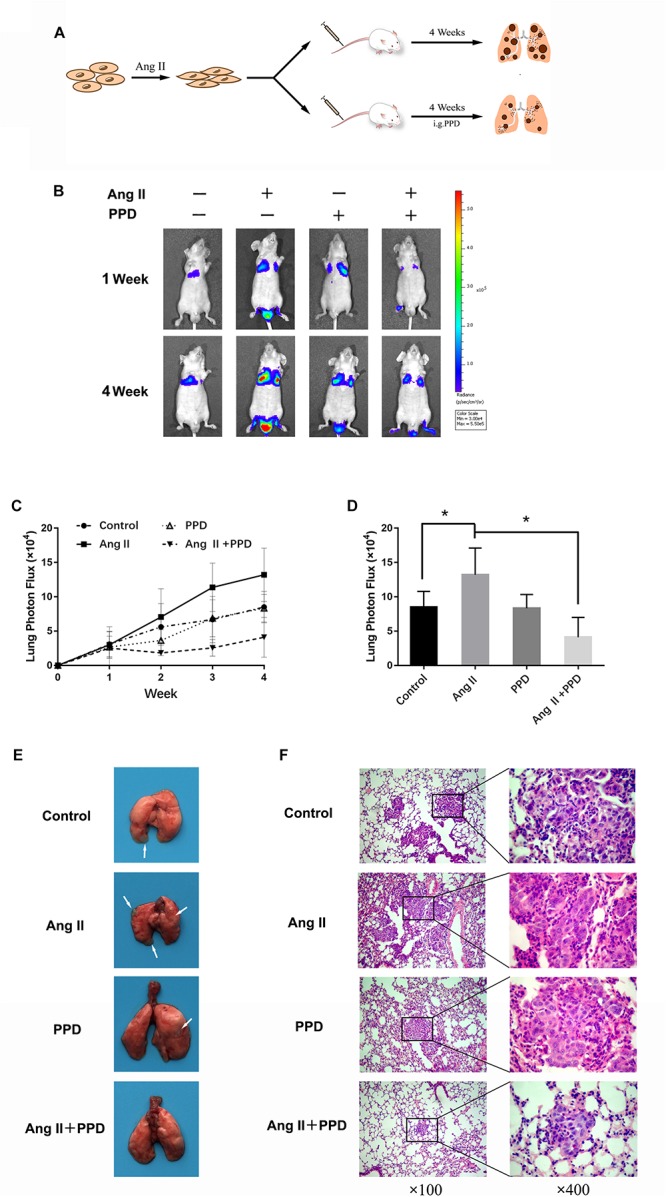
Effectsof PPD on Ang II enhanced lung metastasis *in vivo*. **(A)** Schema of experimental protocol. Nude mice were vein intravenously injected with Ang II-pretreated A549 cells. Subsequently, nude mice were orally administered with the indicated doses of PPD for four consecutive weeks. **(B)** After injection at weeks 1 and 4, the mice were intraperitoneally injected with D-Luciferin for *in vivo* bioluminescence imaging. Tumor metastasis to lungs is shown. **(C)** Mean bioluminescence/time of lung metastasis in xenografted mice, graphed as normalized photon flux/time. **(D)** Mean bioluminescence at 4 weeks. **(E)** Representative images of lung metastatic nodules (arrows refer to tumor lesions). **(F)** Representative pictures of HE staining of the lung issue are shown (magnification, left × 100 and right × 400). *p < 0.05.

Next, we confirmed the antimetastatic activity of PPD using the model described above (Figure 5A). Bioluminescence image analysis showed a remarkable decrease in lung tumor formation in mice treated with PPD compared to in Ang II-pretreated mice (Figures 5B–D). The numbers of the metastatic nodules per lung were substantially diminished by PPD treatment (Figure 5E).

Additionally, histological analysis of the lungs revealed significantly decreased multiplicity and volume of tumor nodules in the PPD-treated mice compared to in Ang II treat mice (Figure 5F). These data clearly indicated that PPD admission has antimetastatic effects on Ang II pretreated A549 cells.

### Involvement of SIRT1 in Ang II-Induced EMT Inhibition by PPD

**FIGURE 6:**
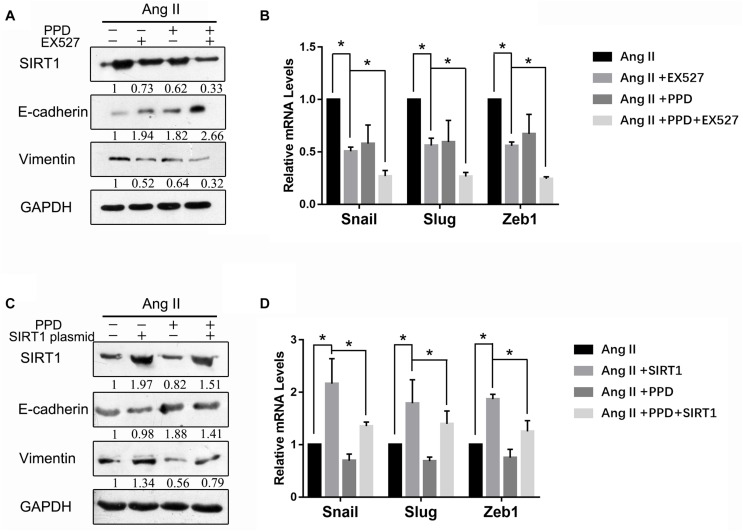
Involvement of SIRT1 in Ang II-induced epithelial-mesenchymal transition (EMT) inhibited by PPD. **(A)** and **(B)** Effect of SIRT1 activation on Ang II-induced EMT. Cells were transfected with SIRT1 plasmid and then further incubated in the presence of PPD for 24 h. The expression of E-cadherin, vimentin, and SIRT1 was determined by western blotting. The mRNA levels of Snail, Slug and Zeb1 were measured by RT-PCR. **(C)** and **(D)** Effect of SIRT1 inhibition on Ang II-induced EMT. Cells were treated with the SIRT1 inhibitor EX-527, and then further incubated in the presence of PPD for 24 h. The expression of E-cadherin, vimentin, and SIRT1 was determined by western blotting. The mRNA levels of Snail, Slug, and Zeb1 were measured by RT-PCR. *p < 0.05.

We next examined the role of SIRT1 in PPD-induced inhibition of EMT driven by Ang II. We first downregulated SIRT1 expression using EX-527, an SIRT1 inhibitor. Figure 6A shows that EX-527 in combination with PPD attenuated Ang II-induced EMT (Figures 6A,B). These results indicate that downregulation of SIRT1 acts synergistically with PPD to inhibit Ang II-induced EMT. We next examined whether Ang II-induced EMT inhibition by PPD can be counteracted by SIRT1 upregulation. We transfected an SIRT1-expressing plasmid into A549 cells. As shown in Figures 6C,D, SIRT1 overexpression markedly increased Ang II-induced EMT, even in the presence of PPD. Taken together, these results indicate that PPD-induced EMT inhibition in NSCLC cells functions through an SIRT1-mediated pathway.

## Discussion

In this study, our results showed that PPD suppresses NSCLC migration and metastasis by inhibiting Ang II-induced EMT, and SIRT1 is involved in PPD-induced EMT inhibition in NSCLC cells. Specifically, Ang II promotes NSCLC migration and metastasis by inducing EMT. Increased expression of SIRT1 in lung cancer plays a distinct role in Ang II-induced EMT. We also investigated the potential of PPD to suppress the migration and metastasis of NSCLC cells after Ang II stimulation. We found that PPD inhibited EMT biomarker changes in response to Ang II, such as E-cadherin, vimentin, Slug, and ZEB1. Additionally, PPD was antagonistic to the increased expression of SIRT1 induced by Ang II. Therefore, SIRT1 may be a therapeutic target for inhibiting cancer metastasis driven by Ang II, and PPD may be an alternative candidate for developing combined targeted therapies antagonistic to Ang II in NSCLC.

EMT is an important step in cancer metastasis (Thiery, 2002). During EMT, epithelial cells often lose their cell-cell adhesion and polarity characteristics and acquire an invasive phenotype. EMT is often characterized by altered expression of proteins including E-cadherin, N-cadherin, and vimentin (Lee et al., 2006). The EMT process is regulated by transcription factors, growth factors, inflammatory cytokines, chemokines, and other enzymes or proteins (Onoue et al., 2006; Asiedu et al., 2011; Yadav et al., 2011). Most components of the RAS including angiotensinogen, angiotensin-converting enzyme, ACE) and angiotensin receptors are expressed locally in a wide variety of tumors, including in lung cancers (George et al., 2010; Okamoto et al., 2012). In animal models, Ang II promoted cancer cell proliferation, migration, and angiogenesis and plays an important role in carcinogenesis and tumor progression in various types of cancer (Chen et al., 2013; Koh et al., 2014). Additionally, an association between Ang II and hematogenous metastasis was reported in several experimental studies (Fujita et al., 2002; Rodrigues-Ferreira et al., 2012). Okamoto et al. reported that Ang II activates intrahepatic cholangiocarcinoma cell migration by mediating the occurrence of EMT (Okamoto et al., 2012). Furthermore, ACE inhibitors that block endogenous Ang II production or ARBs that attenuate Ang II activity efficiently reduced EMT in lung cancer (Maluccio et al., 2001; Miyajima et al., 2002). However, no studies have examined the metastatic effects of Ang II through cancer cell EMT. Our results indicate that Ang II treatment of NSCLC cells promoted cell migration and EMT *in vitro* and triggered rapid development of metastasis in an experimental mice model *in vivo*.

SIRT1, as a class III histone deacetylase family member, regulates many genes by interacting with transcription factors (Michan and Sinclair, 2007; Yamamoto et al., 2007). Some reports indicated that SIRT1 plays a dual role in tumorigenesis as both a tumor suppressor and an oncogene (Simmons et al., 2015), while other reports showed that SIRT1 does not affect carcinogenesis (Boily et al., 2009; Jang et al., 2012). These results indicate that the impact of SIRT1 on EMT varies in various types of cancer and depends on the cellular characteristics and microenvironment of the cell. Ang II is produced primarily in the epithelial cells of the lung and has therapeutic modalities commonly used to treat patients with high blood pressure by enhancing invasion and MMP production in lung cancer cells (Feng et al., 2011; Ishikane and Takahashi-Yanaga, 2018). Therefore, we predicted that a close relationship exists between SIRT1 and Ang II in facilitating EMT. Our data showed increasing expression of SIRT1 in Ang II-treated A549 cells. We also found that EX537, an SIRT1 inhibitor, suppressed EMT induced by Ang II. These results support the hypothesis that SIRT1 plays a key role in the process of EMT induced by Ang II.

PPD, as one of the major active metabolites in ginseng, is the final product of protopanaxadiol saponins following metabolism in the human intestinal flora (Xie et al., 2009). It has been reported that PPD showed broad-spectrum antitumor effects in experimental animals and various cancer cells (Liu et al., 2007; Wang et al., 2008). However, previous studies focused on inhibiting tumor growth, while its role in EMT and metastasis has not been widely examined. Here, we evaluated the ability of PPD to inhibit EMT and metastasis *in vitro* and *in vivo*. PPD treatment resulted in downregulation of E-cadherin expression and upregulation of vimentin expression on NSCLC cells with Ang II. Moreover, our study indicated that PPD significantly decreased tumor metastasis in lung tissues *in vivo*. To further investigate the mechanism of PPD in lung cancer, we evaluated the inhibitory effect of PPD on SIRT1 *in vitro*. We found that SIRT1 was significantly down-regulated by PPD. Our study also indicates that downregulation of SIRT1 by PPD is correlated with EMT in lung cancer cells. Transfected SIRT1 plasmid in A549 cells significantly increased the cell EMT and partially reversed the inhibitory effect of PPD on Ang II-induced EMT. EX-527, a pharmacological inhibitor of SIRT1, and PPD synergistically suppressed Ang II-induced EMT.

In this study, we found that PPD treatment inhibits the migration and metastasis of NSCLC cells driven by Ang II-induced EMT. Further, we found that SIRT1 plays a pivotal role in the inhibitory effect of PPD on Ang II-induced EMT. Accordingly, PPD may be useful for treating NSCLC.

## Ethics Statement

Animals were treated according to the Guide for the Care and Use of Laboratory Animals [United States National Institutes of Health (NIH)] and the Committee for the Care and Use of Laboratory Animals of Jilin University (Changchun, China). The study protocol was approved by the Ethics Committee of Jilin University. Male nude mice were provided by the Experimental Animal Center of Jilin University.

## Author Contributions

YW, HX, and DS carried out most of the studies, participated in the design and coordination of the whole study, and helped in critically revising the draft for important intellectual content. ZL, WF, and MG carried out the cell culture studies, molecular biology experiments, data collection, statistical analysis. XY, MS, and YL participated in the animal experiments, data collection, statistical analysis. XW and WF read and revised the entire manuscript. All authors read and approved the final manuscript.

## Conflict of Interest Statement

The authors declare that the research was conducted in the absence of any commercial or financial relationships that could be construed as a potential conflict of interest.
