# Supplementary material for: 20(S)-Protopanaxadiol Inhibits Angiotensin II-Induced Epithelial- Mesenchymal Transition by Downregulating SIRT1
Source: Front Pharmacol. 2019 May 7;10:475. doi: 10.3389/fphar.2019.00475 (PMC6514190; doi:10.3389/fphar.2019.00475)
Supplement: Supplementary file 1 [file Presentation_1.PPTX]

## Slide 1
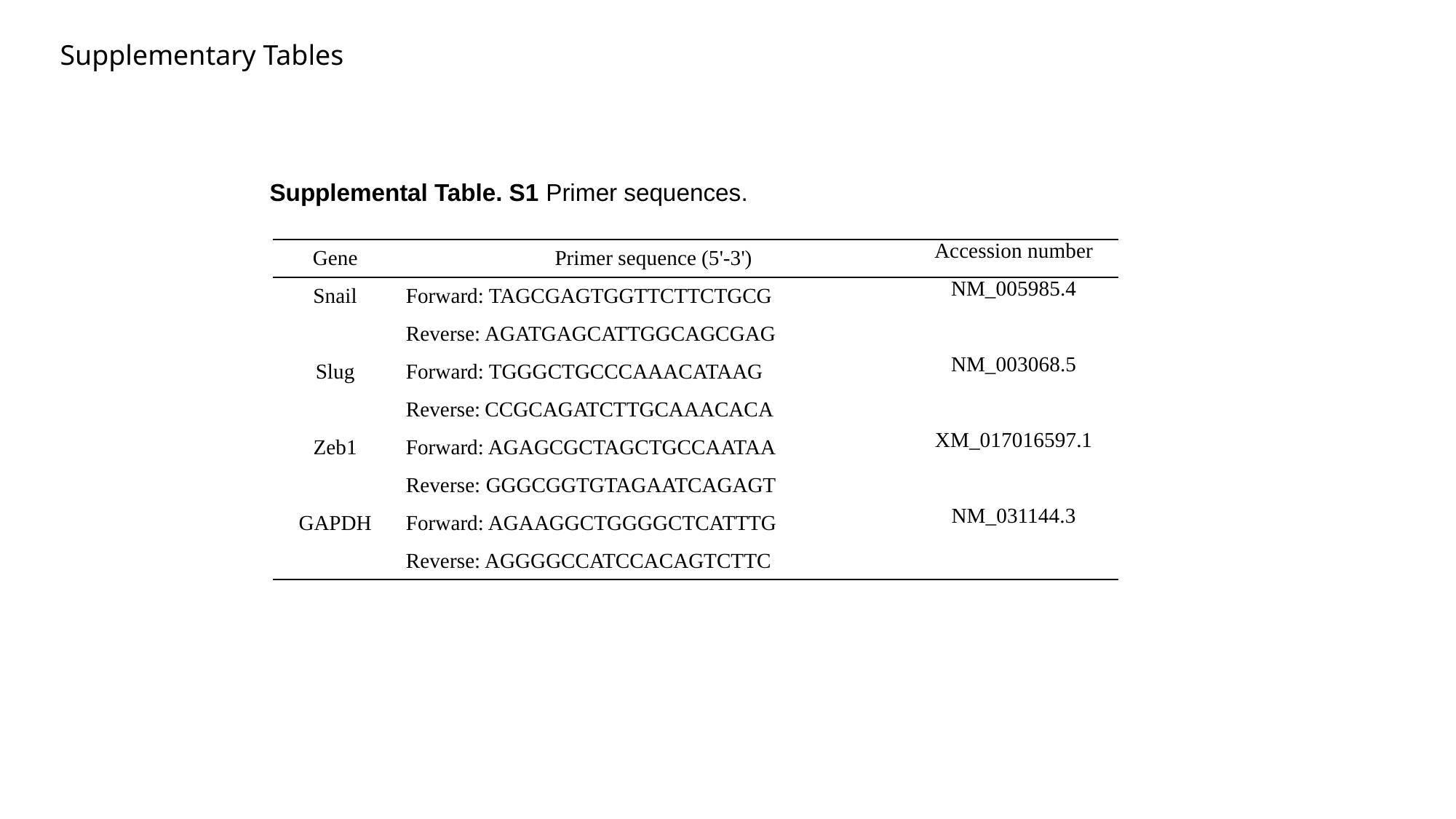

Supplementary Tables
Supplemental Table. S1 Primer sequences.
| Gene | Primer sequence (5'-3') | Accession number |
| --- | --- | --- |
| Snail | Forward: TAGCGAGTGGTTCTTCTGCG | NM\_005985.4 |
| | Reverse: AGATGAGCATTGGCAGCGAG | |
| Slug | Forward: TGGGCTGCCCAAACATAAG | NM\_003068.5 |
| | Reverse: CCGCAGATCTTGCAAACACA | |
| Zeb1 | Forward: AGAGCGCTAGCTGCCAATAA | XM\_017016597.1 |
| | Reverse: GGGCGGTGTAGAATCAGAGT | |
| GAPDH | Forward: AGAAGGCTGGGGCTCATTTG | NM\_031144.3 |
| | Reverse: AGGGGCCATCCACAGTCTTC | |

## Slide 2
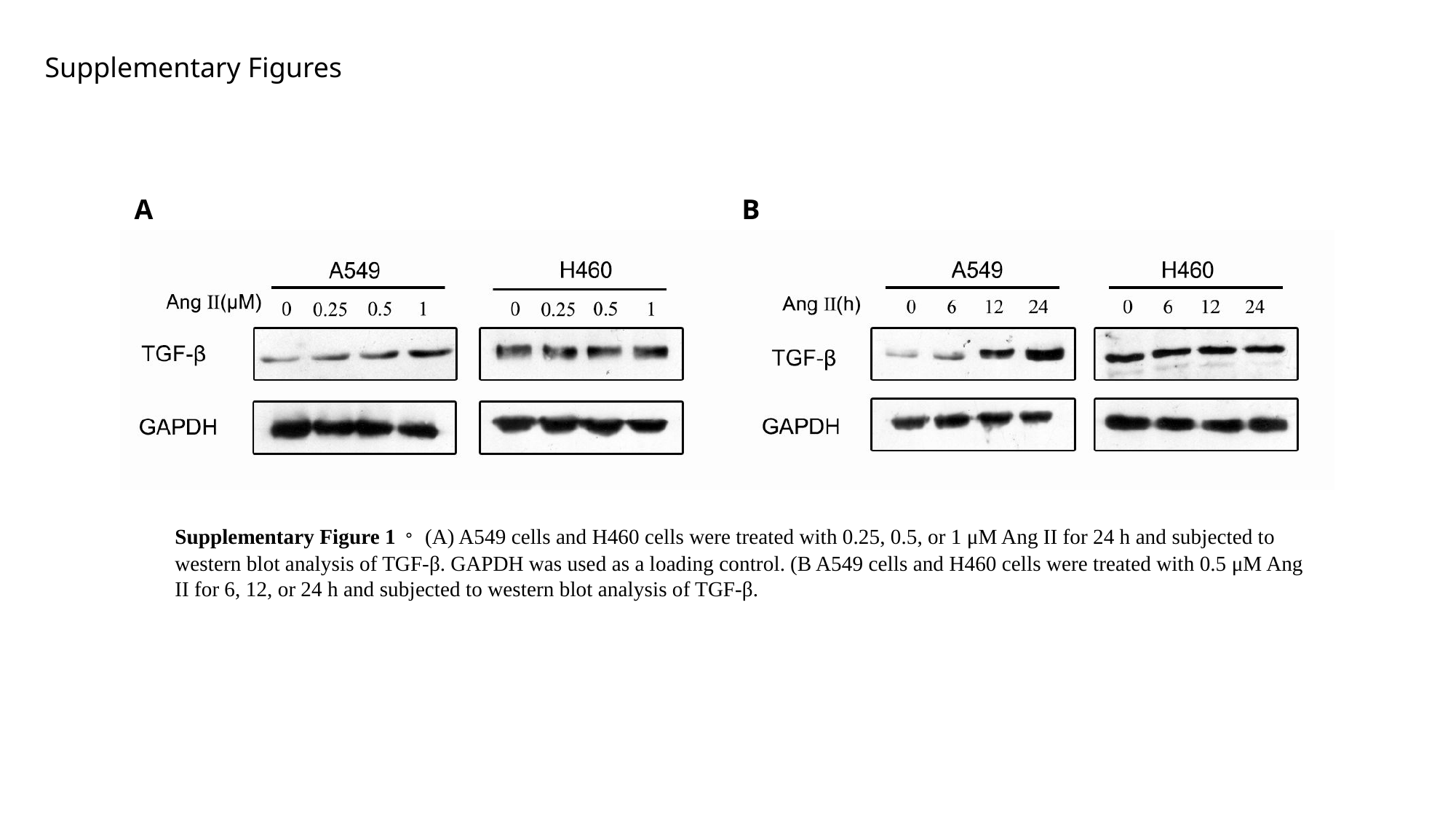

Supplementary Figures
A
B
Supplementary Figure 1。 (A) A549 cells and H460 cells were treated with 0.25, 0.5, or 1 μM Ang II for 24 h and subjected to western blot analysis of TGF-β. GAPDH was used as a loading control. (B A549 cells and H460 cells were treated with 0.5 μM Ang II for 6, 12, or 24 h and subjected to western blot analysis of TGF-β.
